# Astrocytic pathology in Alpers’ syndrome

**DOI:** 10.1186/s40478-023-01579-w

**Published:** 2023-05-31

**Authors:** Laura A Smith, Chun Chen, Nichola Z Lax, Robert W Taylor, Daniel Erskine, Robert McFarland

**Affiliations:** 1 Wellcome Centre for Mitochondrial Research, Faculty of Medical Sciences, Newcastle University, Newcastle upon Tyne, NE2 4HH UK; 2 Translational and Clinical Research Institute, Faculty of Medical Sciences, Newcastle University, Newcastle upon Tyne, NE2 4HH UK; 3 NHS Highly Specialised Service for Rare Mitochondrial Disorders of Adults and Children, Newcastle University, Newcastle Upon Tyne, Newcastle, NE2 4HH UK

**Keywords:** Alpers’ syndrome, POLG, Mitochondrial Epilepsy, Reactive astrogliosis, GFAP, Kir4.1, Aquaporin 4 (AQP4), Glutamine synthetase (GS)

## Abstract

Refractory epilepsy is the main neurological manifestation of Alpers’ syndrome, a severe childhood-onset mitochondrial disease caused by bi-allelic pathogenic variants in the mitochondrial DNA (mtDNA) polymerase gamma gene (*POLG*). The pathophysiological mechanisms underpinning neuronal hyperexcitabilty leading to seizures in Alpers’ syndrome remain unknown. However, pathological changes to reactive astrocytes are hypothesised to exacerbate neural dysfunction and seizure-associated cortical activity in POLG-related disease. Therefore, we sought to phenotypically characterise astrocytic pathology in Alpers’ syndrome. We performed a detailed quantitative investigation of reactive astrocytes in post-mortem neocortical tissues from thirteen patients with Alpers’ syndrome, eight neurologically normal controls and five sudden unexpected death in epilepsy (SUDEP) patients, to control for generalised epilepsy-associated astrocytic pathology. Immunohistochemistry to identify glial fibrillary acidic protein (GFAP)-reactive astrocytes revealed striking reactive astrogliosis localised to the primary visual cortex of Alpers’ syndrome tissues, characterised by abnormal-appearing hypertrophic astrocytes. Phenotypic characterisation of individual GFAP-reactive astrocytes demonstrated decreased abundance of mitochondrial oxidative phosphorylation (OXPHOS) proteins and altered expression of key astrocytic proteins including Kir4.1 (subunit of the inwardly rectifying K^+^ ion channel), AQP4 (astrocytic water channel) and glutamine synthetase (enzyme that metabolises glutamate). These phenotypic astrocytic changes were typically different from the pathology observed in SUDEP tissues, suggesting alternative mechanisms of astrocytic dysfunction between these epilepsies. Crucially, our findings provide further evidence of occipital lobe involvement in Alpers’ syndrome and support the involvement of reactive astrocytes in the pathogenesis of POLG-related disease.

**Supplementary Information:**

The online version contains supplementary material available at 10.1186/s40478-023-01579-w.

## Introduction

Inherited bi-allelic pathogenic variants in *POLG* encoding the catalytic subunit of mitochondrial DNA polymerase gamma (Pol γ), are a common cause of mitochondrial disease presenting with epilepsy [1–3]. *POLG* variants result in inefficient replication of mitochondrial DNA (mtDNA) leading to a loss of mtDNA copy number (mtDNA depletion), particularly within the brain and liver. To date, more than 190 pathogenic variants in *POLG* have been identified [4] and are associated with decreased activity of mitochondrial oxidative phosphorylation (OXPHOS) leading to impaired energy metabolism [2, 5, 6], impacting a range of key mitochondrial signalling events and disturbing cellular function [7].

POLG-related mitochondrial diseases are clinically heterogeneous and can manifest at any age, with an early onset associated with an extremely poor prognosis [8, 9]. Alpers’ syndrome is the most common early-onset form of POLG disease, presenting in infancy, childhood, or adolescence [10–12]. However, young adults harbouring pathogenic *POLG* variants may present with a similar clinical phenotype, highlighting the continuum of POLG diseases [10].

Refractory epilepsy is a distinguishing early feature of Alpers’ syndrome and is characterised by focal-onset seizures in the primary visual cortex, though the epilepsy may evolve to include myoclonic seizures, status epilepticus or *epilepsia partialis continua* [13–15]. Stroke-like episodes, which are acute transient periods of neurological deterioration, are a common manifestation of Alpers’ syndrome and are hypothesised to be driven by focal seizure activity [10, 16]. Patients with Alpers’ syndrome also present with neurodevelopmental delay or regression, cortical blindness, cerebellar ataxia and hepatic failure [12, 17].

Epilepsy in Alpers’ syndrome is closely associated with severe cortical degeneration and encephalopathy [12]. The mechanisms underpinning POLG-related epilepsy are yet to be fully elucidated, however, recent neuropathological studies demonstrate an involvement of gamma aminobutyric acid (GABA)-ergic inhibitory interneurons [18–20]. Degeneration of cortical interneurons, in particular occipital parvalbumin-expressing interneurons, accompanied by decreased expression of mitochondrial OXPHOS proteins within residual interneurons, is thought to underlie impaired inhibitory neurotransmission leading to neuronal network hyperexcitability [19]. Diminished firing of fast-spiking interneurons in response to pharmacological inhibition of mitochondrial OXPHOS complexes I and IV has also been demonstrated in rodent hippocampal slices, highlighting the vulnerability of interneurons to mitochondrial dysfunction [21]. However, despite diminished fast-spiking interneuron activity, interictal activity was not induced suggesting a concomitant pathological process drives mitochondrial-associated seizures.

Reactive astrogliosis, characterised by the presence of abnormal hypertrophic astrocytes enriched within regions of focal neuronal loss, is a common pathological feature of Alpers’ syndrome and POLG disease [6, 17]. Cortical reactive astrocytes in patients with adult-onset mitochondrial disease, including patients with POLG-related epilepsy, demonstrate mitochondrial OXPHOS protein deficiencies and decreased abundance of the ATP-dependent enzyme glutamine synthetase [22]. Since mitochondrial dysfunction impairs the capacity of astrocytes to respond appropriately to neuronal insults, it is reasonable to surmise that astrocytes may play an important role in the aetiology of Alpers’ syndrome [23, 24]. In support of this hypothesis it has been demonstrated that inhibition of astrocytic aconitase by fluorocitrate, in conjunction with OXPHOS complex I and complex IV impairment, induced spontaneous interictal activity in in vitro brain slices [22].

Astrocytic dysfunction has also been implicated in the pathogenesis of more common epilepsies including temporal lobe epilepsy (TLE). The loss of essential astrocytic proteins including glutamine synthetase, which metabolises the excitatory neurotransmitter glutamate, and Kir4.1, a subunit of inwardly rectifying potassium channels which functions to remove excess K^+^ ions from the synapse, appears to promote neuronal hyperexcitability in TLE [25–29]. The distribution and expression of aquaporin-4 (AQP4), an astrocytic-specific water channel, is also frequently altered in patients with epilepsy. AQP4 is thought to alter ionic flux and the volume of brain parenchyma thus modulating seizure susceptibility [28, 30–32].

Elucidating the role of reactive astrocytes in Alpers’ syndrome is not only critical to better understand the contribution of astrocytic pathology to neurodegeneration and neural dysfunction in this condition, but also to identify shared pathomechanisms with other epilepsies. Improved understanding of these pathological processes will, in turn, inform the development of specific model systems of POLG-related pathology; an important prerequisite for therapeutic development. For these reasons, we sought to identify whether astrocytes in Alpers’ syndrome and POLG-related disease manifest morphological changes, mitochondrial OXPHOS protein deficits, and/or alterations to glutamate metabolism and ionic homeostasis that could contribute to the neurological phenotype.

## Materials and methods

### Patient cohort

Formalin-fixed paraffin-embedded (FFPE) post-mortem brain tissues were obtained from seven patients with clinically- and neuropathologically-defined Alpers’ syndrome, three patients with genetically-confirmed Alpers’ syndrome, and three young adult patients with POLG-related encephalopathy (Table 1). All patients were grouped together for analyses and are referred to as patients with Alpers’ syndrome from hereafter. Since seizures in Alpers’ syndrome often have an occipital focus, brain tissue was obtained from the primary visual cortex (Brodmann Area 17) and was compared to the frontal cortex (Brodmann Area 9), which typically has less involvement in Alpers’ syndrome. Brain tissue from patients with Alpers’ syndrome was compared to that from 8 neurologically-normal controls and 5 sudden unexpected death in epilepsy (SUDEP) patients, which were included to control for generalised epilepsy-associated pathology (Supplementary Tables 1 and Supplementary Table 2). SUDEP cases were defined as a premature unexplained death of an epilepsy patient for which there was no cause of death identified at autopsy [33]. The cohort was matched for age at death (Kruskal-Wallis, *p* = 0.215), sex (*X*^2^ test, *p* = 0.756), post-mortem interval (Kruskal-Wallis, *p* = 0.168) and duration of formalin fixation (Kruskal-Wallis, *p* = 0.563). Ethical approval for the use of these tissues was provided by the respective brain banks (Supplementary Table 1).

**Table 1:** Alpers’ syndrome patient cohort

*Patient*:	*Sex*:	*Age at onset*:	*Age at death*:	*Bi-allelic pathogenic * *POLG variants*:		*Publications*:
				*cDNA*	*Protein*	
Pt.01^†^	M	2 m	5.5 m	Unknown	Unknown	[18, 19]
Pt.02^†^	M	4 m	13 m	Unknown	Unknown	[18, 19]
Pt.03	F	11 m	14 m	c.1399G > A/ c.2542G > A	p.[Ala467Thr]/ p.[Gly848Ser]	[18, 19]
Pt.04^†^	M	6 m	17 m	Unknown	Unknown	[19]
Pt.05^†^	F	12 m	18 m	Unknown	Unknown	[19]
Pt.06^†^	M	18 m	2.8 y	Unknown	Unknown	[19]
Pt.07	F	6 m	7 y	c.2243G > C/ c.2243G > C/	p.[Trp748Ser]/ p.[Trp748Ser]	[18, 19, 34]
Pt.08	M	2 y	11.9 y	c.1399G > A/ c.2542G > A	p.[Ala467Thr]/ p.[Gly848Ser]	[19]
Pt.09^†^	M	6 y	12.5 y	Unknown	Unknown	[18, 19]
Pt.10^†^	F	6 m	14 y	Unknown	Unknown	[18, 19]
Pt.11	F	18 y	23 y	c.1399G > A/ c.1399G > A	p.[Ala467Thr]/ p.[Ala467Thr]	[19, 35]
Pt.12	F	20 y	24 y	c.1399G > A/ c.2243G > C	p.[Ala467Thr]/ p.[Trp748Ser],	[19, 20, 22, 36–39)
Pt.13	F	16 y	28 y	c.1399G > A/ c.2243G > C	p.[Ala467Thr]/ p.[Trp748Ser]	[19]

Abbreviations: *m* months; *y* years; *F* female; *M* male. Clinical and neuropathological details for the patients with Alpers’ syndrome (Pt.01 – Pt.10) and adult patients with POLG-related encephalopathy (Pt.11 – P13) are summarised in [19]. Historical patients (†) remain without a molecular diagnosis since they precede identification of pathogenic *POLG* variants known to cause Alpers’ syndrome and extraction of DNA from FFPE tissues to sequence *POLG* was unsuccessful. *POLG* RefSeq: NM_002693

### Immunohistochemistry for identification of reactive astrocytes

Immunohistochemistry was performed to identify GFAP-reactive(+) astrocytes in 5µm-thick sections as previously described [19, 38]. Briefly, this involved heat-mediated antigen retrieval using 10mM trisodium citrate (pH 6.0), blocking in 3% hydrogen peroxide (H_2_O_2_) and incubating sections with a glial fibrillary acidic protein (GFAP) antibody, optimally diluted (1:15,000) in Tris-buffered saline, 0.1% Tween 20® (TBST), overnight at 4^o^C (Supplementary Table 3). The Menarini Diagnostics horseradish-peroxidase polymer kit was used for primary antibody amplification which was visualised using 3,3’-diaminobenzadine (DAB) chromogen. Sections were counterstained with Mayer’s Haematoxylin.

### Quantification of reactive astrogliosis labelling

GFAP-stained chromogen sections were imaged using an Olympus BX51 stereology brightfield microscope. An area of at least 5mm^2^, encompassing all cortical layers, was outlined at x2 magnification and images were captured within the contour at x20 magnification using the Meander scan function in Stereo Investigator (MBF Bioscience). To detect the GFAP-DAB + stain, an Open Source Plugin for analysis of immunohistochemistry sections was used in FIJI (Supplementary Fig. 1) [40]. Images were converted to 8-bit and a threshold was set to label all GFAP-DAB + structures. The percentage area of GFAP + labelling was measured per image, and averaged per case, to provide an indication of the severity of reactive astrogliosis.

### Multiplex immunofluorescence

To explore changes to the mitochondrial OXPHOS system within reactive astrocytes, a previously optimised quadruple immunofluorescence protocol was performed [22]. Sections were co-stained with GFAP, NADH:ubiquinone oxidoreductase subunit B8 (NDUFB8; nuclear DNA-encoded mitochondrial complex I subunit), cytochrome *c* oxidase I (COXI; complex IV subunit) and porin (VDAC1; mitochondrial mass marker) (Supplementary Table 3). NDUFB8 and COXI protein expression are known to be associated with the structure and function of OXPHOS complex I and complex IV, respectively [41, 42], and a loss of these subunits have previously been reported in inhibitory interneurons in this Alpers’ syndrome patient cohort and astrocytes from adult patients with mitochondrial epilepsy, indicative of enzymatic OXPHOS deficiencies [18, 19, 22].

To evaluate changes to key astrocytic proteins which are frequently altered in epilepsy, with the aim of identifying whether pathological changes in Alpers’ syndrome are typical of alterations associated with epilepsy generally, separate sections were stained with antibodies raised against Kir4.1 (potassium ion channel subunit), AQP4 (astrocytic water channel) and glutamine synthetase (glutamate metabolising enzyme), using GFAP and Hoechst (1:1,200 dilution, nuclear marker) to identify individual astrocytes (Supplementary Table 3).

The immunofluorescence protocol briefly involved heat-mediated antigen retrieval in 1mM EDTA (pH 8.0) or 10mM trisodium citrate (pH 6.0), followed by blocking in 10% normal goat serum (NGS) for 1 h at room temperature. Sections stained with NDUFB8 were also blocked in avidin and biotin for 15 min each. Primary antibodies were diluted in TBST and were incubated overnight at 4^o^C. To amplify the NDUFB8 signal, sections were incubated with a biotinylated mouse IgG1 antibody diluted in 10% NGS-TBST for 30 min at room temperature. Appropriate Alexa-Fluor conjugated secondary antibodies were then applied to the sections for 2 h at 4^o^C (1:100 dilution, Supplementary Table 4). If there was a spare channel available on the antibody panel, sections were incubated with Hoechst for 15 min at room temperature. To minimise autofluorescence, sections were suspended in 3.0% Sudan Black B prior to being mounted in ProLong™ Gold antifade reagent. Only tissues fixed in formalin for less than one year were included for immunofluorescence experiments, since long fixation has a detrimental effect on antigenicity [43].

### Confocal microscopy

An inverted ZEISS LSM800 confocal microscope and ZEISS ZEN (blue edition) software were used to image immunofluorescent sections at x63 magnification as previously described [19], with the addition of Airyscan detection to increase resolution [44]. Astrocytes were randomly selected for image capture across all cortical layers in Brodmann Area 17 and 9, based on identification of GFAP + astrocytic cell bodies and associated processes. To image mitochondria within individual astrocytes and their processes, z-stacking was performed; astrocytes were imaged on the x-, y- and z-planes using a z-step size of 0.21 μm. Optimised image capture settings were maintained for all cases, per experiment. Approximately 40 astrocytes were imaged for two-dimensional analysis of Kir4.1, AQP4 and glutamine synthetase, and approximately 20 three-dimensional astrocytes were imaged for assessment of mitochondrial markers per case, due to the increased duration of image capture.

### Two-dimensional analysis of astrocytes

Using ZEISS ZEN Blue Desk software, astrocytes and their processes were automatically outlined by setting an intensity threshold for the GFAP + channel and the area of each astrocyte was recorded. Within each astrocyte, the mean optical intensity of all markers were measured.

### Three-dimensional analysis of mitochondria in astrocytes

Z-stacked astrocytes were analysed using Volocity® software. Astrocytes were detected and set as an ‘object’ based on the GFAP + signal labelling astrocytic cell bodies (> 50µm^3^) and their processes. The mean optical intensities of all channels were measured within the astrocytic object, and the total volume (µm^3^) of each astrocyte was quantified. The intensity values of NDUFB8 and COXI were log-transformed and normalised to log-transformed porin intensity, as previously described [19]. This was to ensure altered OXPHOS subunit expression was not due to changes in overall mitochondrial mass. Z-scores were calculated using control NDUFB8/Porin and COXI/Porin ratios to determine the severity of NDUFB8 and COXI deficiencies in Alpers’ syndrome patient astrocytes, based on standard deviation (SD) limits [45]. To make inferences about changes to mitochondrial mass in Alpers’ syndrome astrocytes, z-scores were also calculated using the log-transformed porin intensity data.

### Statistical analysis

Statistical analyses were performed using GraphPad Prism 9.0 (GraphPad Software, Inc., La Jolla, California) and R (R Core Team, 2020). Normality of data was assessed using the Shapiro-Wilk test and Q-Q plots were visually inspected. To identify changes to astrocytes from individual patients with Alpers’ syndrome, astrocytes were pooled per patient and compared to pooled astrocytes from the control group and SUDEP patient group using the Kruskal-Wallis test followed by Dunn’s method for multiple comparison. To statistically compare changes to astrocytes in focal lesioned cortex compared to adjacent non-lesioned cortex (for Patient 11, Patient 12 and Patient 13), and to compare occipital versus frontal cortical astrocytes (for patients for which tissues were available for both cortical regions), the Mann-Whitney *U* test was used. Group level analyses were also performed using a linear mixed effects model adjusted for multiple comparisons [46], accounting for the total number of cells analysed per case within each group. For correlation analyses, z-scores were calculated for the percentage area of GFAP + labelling and were compared to our published neuronal density data set (expressed as z-scores) [19] using the Spearman-rank test. The level of significance was set at α = 0.05 for all analyses.

## Results

### Clinical and neuropathology details

The patient cohort consisted of ten patients with clinically- and neuropathologically-defined Alpers’ syndrome, including three patients with confirmed bi-allelic pathogenic variants in *POLG*, and three adult patients with POLG-related encephalopathy (Table 1). Refractory epilepsy was the main presenting symptom in all patients and stroke-like episodes were confirmed radiologically and neuropathologically in the primary visual cortex of three patients (Patient 11, Patient 12 and Patient 13; Supplementary Table 2) [19]. Developmental delay and/or developmental regression, visual impairment including cortical blindness, ataxia and hepatic dysfunction were also common in this patient cohort [19].

Post-mortem neurodegenerative changes frequently showed a predilection for the occipital cortex and included diffuse neuronal loss and severe thinning of the cortical ribbon. Focal necrotic lesions identified in occipital tissues from Patient 11, Patient 12 and Patient 13 were characterised by demarcated regions of almost total neuronal loss, marked spongiosis, severe reactive astrogliosis and microglial activation. Although gliotic changes were identified in most Alpers’ syndrome patient tissues, reactive astrogliosis was noticeably more severe in the occipital cortex compared to other cortices. To determine the involvement of astrocytes in the pathophysiology of Alpers’ syndrome and POLG-related disease, we sought to phenotypically characterise reactive astrocytes within occipital cortex tissues.

### Reactive astrogliosis is severe in the occipital cortex

**Fig. 1:**
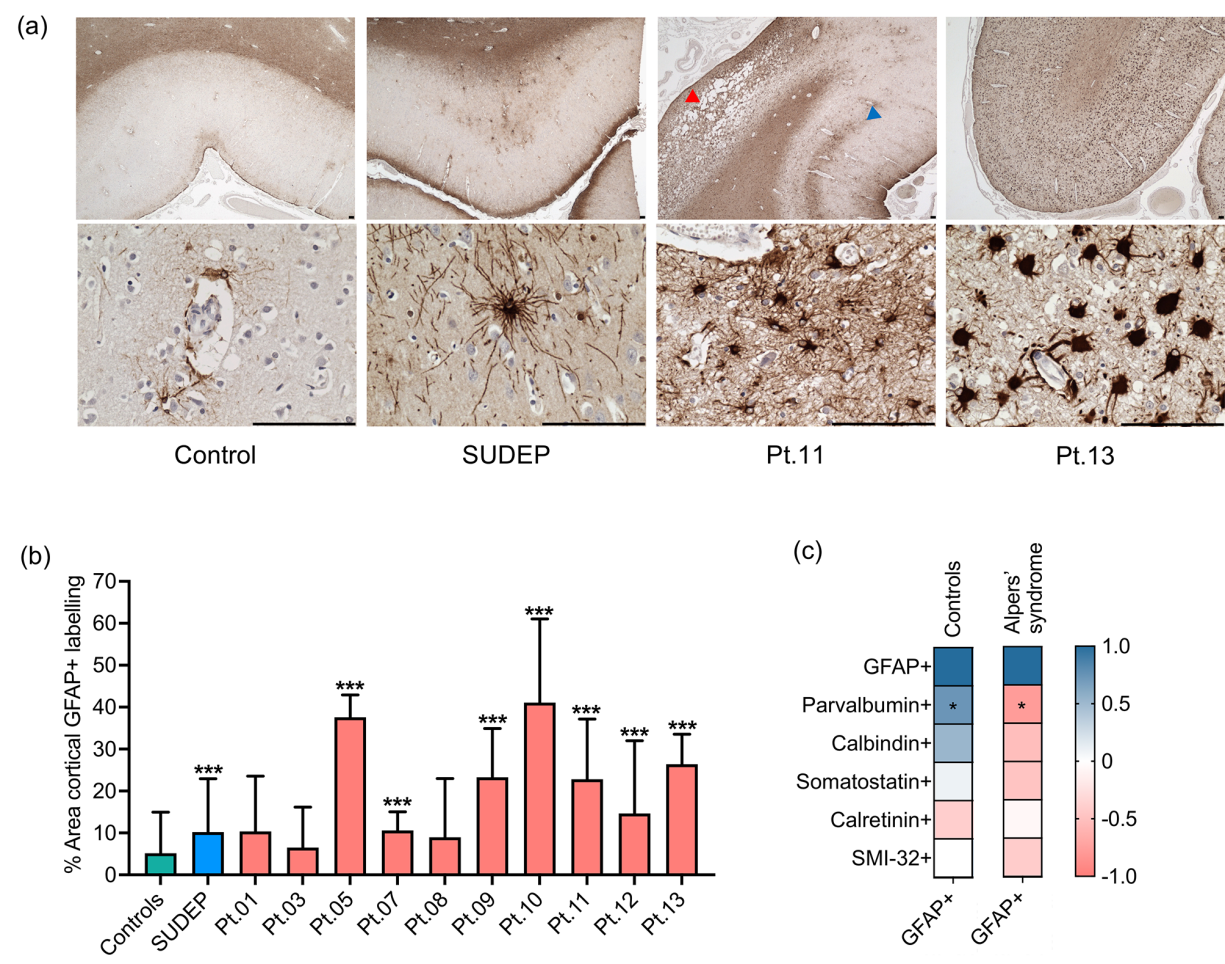
Reactive astrogliosis in Alpers’ syndrome. **(a)** Increased intensity and distribution of glial fibrillary acidic protein (GFAP) + labelling in occipital cortex tissues from patients with Alpers’ syndrome (Pt.11 and Pt.13) relative to controls and SUDEP patients. GFAP + labelling is intense in the necrotic lesion of Pt.11 (red arrow head), and shows selective cortical layer involvement in adjacent preserved tissue (blue arrow head). Hypertrophic ameboid GFAP + astrocytes are abundant across all cortical layers of Pt.13. Scale bars = 100 μm. **(b)** Quantitative analysis of the percentage area of cortical GFAP + staining revealed significantly increased GFAP + labelling in multiple Alpers’ syndrome patient tissues compared to controls (N = 8) [40]. Data presented as mean + SD. Multiple comparison analyses relative to control data: ** *P* < 0.01, *** *P* < 0.001. (**c**) Analyses assessing the correlation between the percentage area of GFAP + labelling with the density of interneuron subtypes (parvalbumin+, calretinin+, calbindin + and somatostatin+) and pyramidal neurons (SMI-32+), expressed as z-scores, using our published neuronal density data set [19]. Legend = Spearman-rank r value. * *P* < 0.05, ** *P* < 0.01

Most occipital cortex tissues from patients with Alpers’ syndrome showed a visible increase in GFAP + labelling relative to control tissues, including an apparent increased density and intensity of GFAP + cells. Alpers’ syndrome patient astrocytes also frequently appeared hypertrophic, characterised by swollen cell bodies and shortening or a lack of processes (Fig. 1). Reactive astrogliosis in Alpers’ syndrome tissues frequently involved all cortical layers and was particularly severe in areas of severe neurodegeneration. However, some Alpers’ syndrome patient tissues demonstrated selective cortical layer involvement (Fig. 1).

Our qualitative observations of reactive astrogliosis in the occipital cortex were confirmed by a higher mean percentage area of GFAP + labelling in seven of ten patients with Alpers’ syndrome relative to controls (*P* < 0.05). The SUDEP patient group also demonstrated increased GFAP + labelling relative to controls (*P* < 0.01); this frequently involved increased ramification of astrocytic processes, rather than an apparent increased density of astrocytes (Fig. 1), which was distinct to the numerous ameboid astrocytes observed in Alpers’ syndrome tissues.

We next compared the severity of reactive astrogliosis with the loss of specific neuronal subtypes in Alpers’ syndrome patient tissues using our published neuronal density data [19]. Interestingly, reactive astrogliosis negatively correlated with parvalbumin + interneuron density in the occipital cortex of patients with Alpers’ syndrome (r = -0.78, *P* = 0.017), but this was not observed with any other interneuron subtype quantified (Fig. 1c). This suggests that parvalbumin + interneuron loss in the visual cortex is selectively accompanied by reactive astrogliosis in Alpers’ syndrome.

### Hypertrophy of occipital astrocytes

**Fig. 2:**
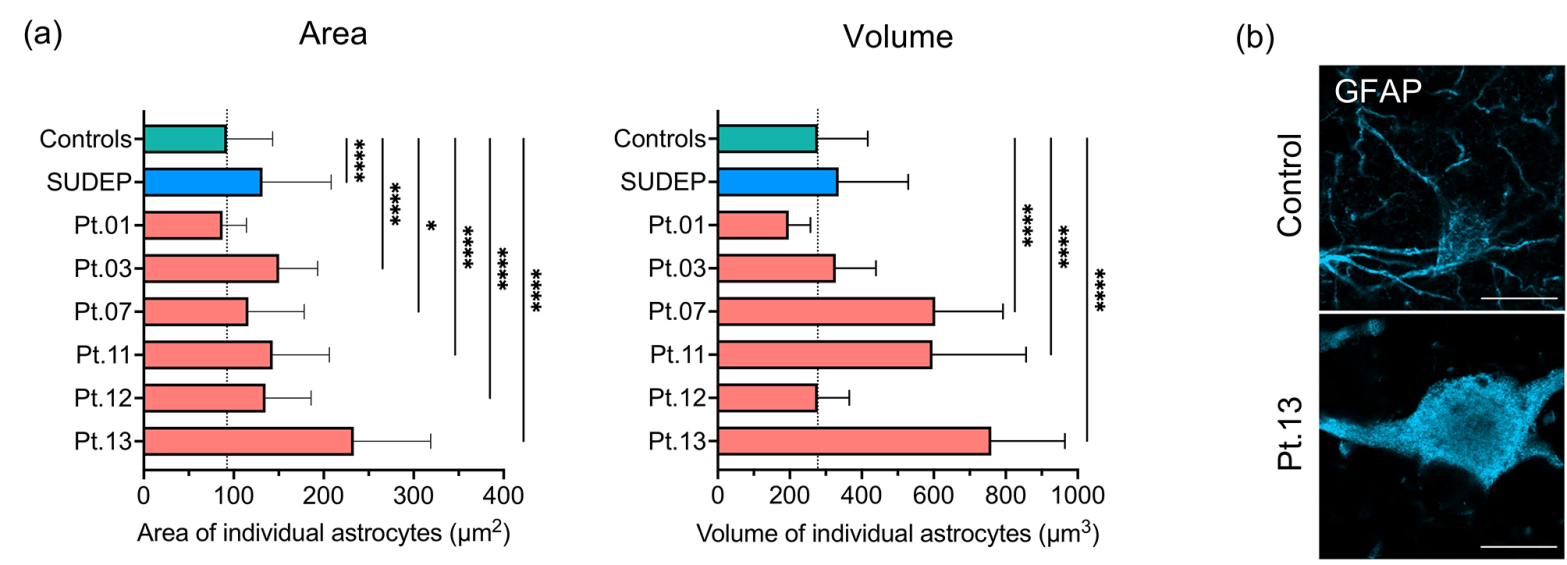
Hypertrophic astrocytes in Alpers’ syndrome. **(a)** The mean (+ SD) area (µm^2^) and volume (µm^3^) of individual two-dimensional (2-D) and three-dimensional (3-D) GFAP + astrocytes, respectively, are increased in occipital cortex tissues from patients with Alpers’ syndrome relative to control astrocytes (N = 5 control cases). Dotted line indicates the mean area and volume of control astrocytes. Random individual 2-D and 3-D astrocytes were imaged for analysis. Multiple comparison analyses relative to control data: * *P* < 0.05, ** *P* < 0.01, **** *P* < 0.0001. **(b)** Hypertrophic astrocytes identified based on GFAP + staining of Patient 13 are presented. Scale bars = 10 μm

Since reactive astrocytes from Alpers’ syndrome patient occipital tissues frequently appeared enlarged (Fig. 1), the size of GFAP + astrocytes identified using immunofluorescence were measured to confirm this visual observation. Analysis revealed a signifincantly increased surface area (µm^2^) of two-dimensional astrocytes from most patients with Alpers’ syndrome (*P* < 0.05), and half of patients demonstrated a significantly increased volume (µm^3^) of z-stacked GFAP + astrocytes compared to control occipital astrocytes (*P* < 0.0001) (Fig. 2). These data suggest hypertrophy of astrocytes occurs frequently in the occipital cortex in Alpers’ syndrome.

### Mitochondrial OXPHOS protein levels in occipital astrocytes

To determine whether astrocytes have altered expression of mitochondrial OXPHOS subunits in Alpers’ syndrome, the intensity of NDUFB8 and COXI immunofluorescence signals, normalised to porin, were quantified within GFAP + astrocytes in occipital cortex tissues from six patients with Alpers’ syndrome [45].

**Fig. 3:**
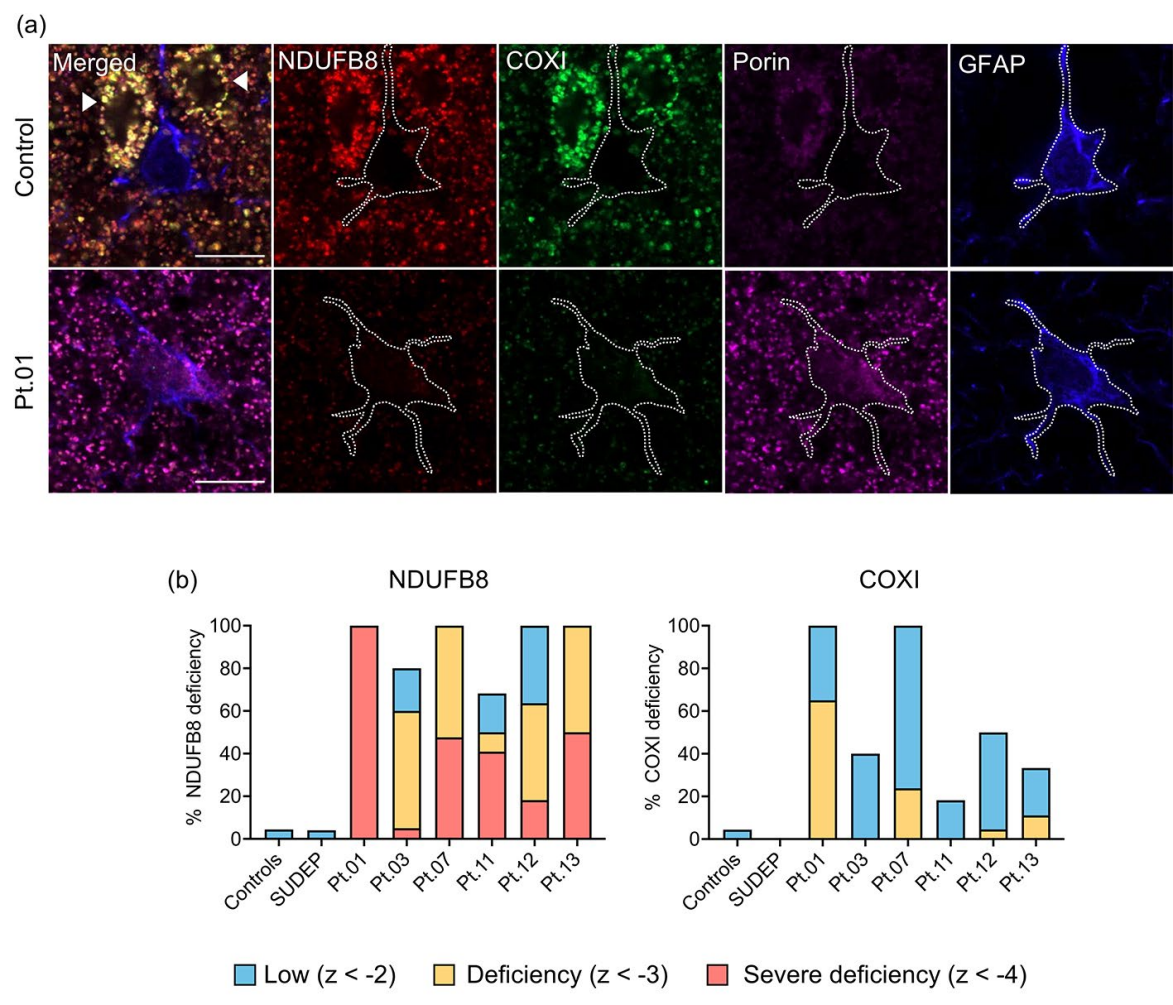
Oxidative phosphorylation protein deficiencies in Alpers’ syndrome patient astrocytes. **(a)** Quadruple immunofluorescence assay revealed decreased protein abundance of NDUFB8 (complex I subunit) and COXI (complex IV subunit) within GFAP + astrocytes in occipital cortex tissues from patients with Alpers’ syndrome relative to control astrocytes, despite increased porin signal (mitochondrial marker). Note astrocytes demonstrate weak immunoreactivity of all mitochondrial markers compared to adjacent neurons (arrow head). Scale bars = 10 μm. **(b)** Graphs demonstrate the percentage of GFAP + astrocytes with decreased mean optical intensity of NDUFB8 and COXI normalised to porin, relative to control astrocytes. Levels of deficiencies are based on standard deviation limits of control data: z-score < -2 = low expression; z-score < -3 = deficient; z-score < -4 = severely deficient [45]. N = 5 Controls, N = 5 SUDEP cases

The immunoreactivity of NDUFB8 and COXI was variably decreased across Alpers’ syndrome patient tissues, despite visible levels of porin (Fig. 3a). Quantification of the mean optical intensity of the mitochondrial markers within GFAP + astrocytes revealed the Alpers’ syndrome patient group demonstrated significantly low (z-score < -2) to severely deficient (z-score < -4) levels of NDUFB8, and to a lesser extent COXI, compared to control astrocytes (*P* < 0.001) (Fig. 3b). However, the levels of NDUFB8 and COXI normalised to porin were normal within GFAP + astrocytes from SUDEP patients (*P* > 0.05).

Since increased mitochondrial mass has previously been reported to accompany OXPHOS deficiencies within parvalbumin + interneurons in this patient cohort [19], the intensity of porin (expressed as a z-score) within GFAP + astrocytes was analysed. Group level analysis revealed no difference in the intensity of porin in the Alpers’ syndrome patient astrocytes relative to the control group (*P* > 0.05). However, analysis of individual patients with Alpers’ syndrome demonstrated a significantly increased porin intensity in four of six patients (*P* < 0.01), with a trend towards increased porin in focal lesioned cortex versus adjacent non-lesioned cortex (*P* < 0.001) (Supplementary Fig. 2).

### Increased expression of Kir4.1 and AQP4 proteins in occipital astrocytes

Kir4.1 is reported to be downregulated in patients with epilepsy which may impair the capacity of astrocytes to buffer extracellular K^+^ ions and thereby promote seizure-associated activity [29]. To investigate whether similar changes to Kir4.1 protein expression occur in Alpers’ syndrome, the mean optical intensity of Kir4.1 was measured within individual GFAP + astrocytes.

**Fig. 4:**
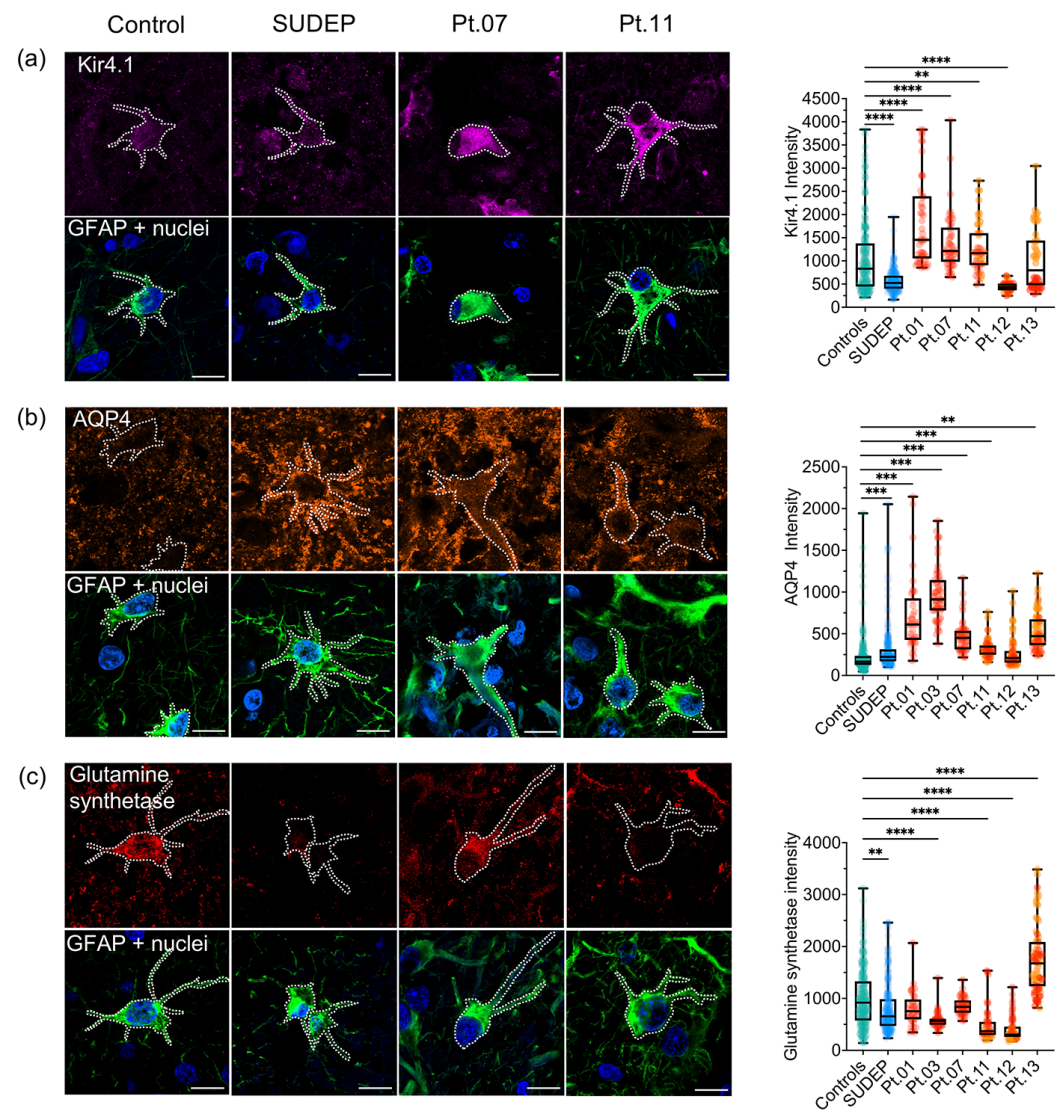
Altered expression of Kir4.1, AQP4 and glutamine synthetase in Alpers’ syndrome patient astrocytes. **(a)** Immunofluorescence to identify Kir4.1 (purple), **(b)** AQP4 (orange) and **(c)** glutamine synthetase (red), using GFAP+ (green) and Hoescht (blue, nuclear marker) to identify individual reactive astrocytes in the occipital cortex. Scale bars = 10 μm. The mean optical intensity of Kir4.1, AQP4 and glutamine synthetase within individual GFAP + astrocytes are presented as circles. For Pt.11 – Pt.13 occipital cortex data, orange circles denote astrocytes imaged in lesioned cortex, and red circles denote astrocytes imaged in adjacent non-lesioned cortex. Box plots demonstrate the median, upper/lower quartiles and range of data. Multiple comparison analyses relative to control data: *P < 0.05, ** *P* < 0.01, *** *P* < 0.001, **** *P* < 0.0001. Note: Kir4.1 intensity was selectively increased in astrocytes from lesioned cortex of Pt.13 (*P* < 0.0001). N = 5 controls, N = 5 SUDEP

Group level analysis revealed no difference in the intensity of Kir4.1 in pooled astrocytes from the Alpers’ syndrome patient group compared to control and SUDEP patient groups (*P* > 0.05). However, interestingly analysis of individual patients with Alpers’ syndrome revealed a significant increase in Kir4.1 intensity in astrocytes from four of five patients with Alpers’ syndrome relative to control astrocytes (*P* < 0.01) (Fig. 4a), albeit the intensity of Kir4.1 was significantly increased selectively in lesioned occipital cortex of Patient 13 (*P* < 0.0001) (Supplementary Fig. 3). Consistent with findings in TLE, the SUDEP patient group demonstrated a significant decreased intensity of Kir4.1 in occipital astrocytes compared to controls and patients with Alpers’ syndrome (*P* < 0.05), suggesting a distinct phenotypic change to astrocytes in Alpers’ syndrome versus SUDEP.

Since the expression of Kir4.1 is reported to be tightly coupled to the expression of AQP4 [31, 47], immunofluorescence to identify the astrocytic water channel within GFAP + astrocytes was performed. Group level analysis revealed a significant increase in the intensity of AQP4 in the Alpers’ syndrome patient group compared to the control group in the occipital cortex (*P* = 0.03). Comparison of individual patients with Alpers’ syndrome to the control group also revealed a significant increased intensity of AQP4 in five of six patients with Alpers’ syndrome (*P* < 0.01), who showed increased levels of Kir4.1 in previous analyses (Fig. 4b). The intensity of AQP4 was also significantly increased in occipital astrocytes from the SUDEP patient group compared to control astrocytes (*P* < 0.0001), albeit the intensity of AQP4 was significantly higher in most patients with Alpers’ syndrome versus the SUDEP patients (*P* < 0.05). These data suggest astrocytes of the occipital cortex frequently show an increase in Kir4.1 and AQP4 protein expression in Alpers’ syndrome compared to control and SUDEP patient astrocytes.

### Altered levels of glutamine synthetase

The protein levels of glutamine synthetase have been reported to be decreased in adult patients with mitochondrial epilepsy [22], and patients with temporal lobe epilepsy [25]. To evaluate whether similar changes occur in Alpers’ syndrome, the mean optical intensity of glutamine synthetase was quantified within GFAP + astrocytes.

Group level analysis revealed no statistical difference between the intensity of glutamine synthetase in pooled astrocytes from the Alpers’ syndrome patient group compared to the control and SUDEP patient groups (*P* > 0.05). However, comparison of astrocytes from individual patients with Alpers’ syndrome relative to the control group revealed significantly altered intensity of glutamine synthetase in occipital astrocytes in four of six patients with Alpers’ syndrome (*P* < 0.0001) (Fig. 4c). This included a significantly decreased intensity of glutamine synthetase in occipital astrocytes in three of six patients with Alpers’ syndrome (*P* < 0.0001), which was more severe in focal lesioned versus non-lesioned cortex (*P* < 0.0001) (Supplementary Fig. 3). However, surprisingly the intensity of glutamine synthetase was significantly increased in occipital astrocytes from Patient 13 (*P* < 0.0001), including a more severe increase in focal lesioned versus non-lesioned cortex (P < 0.01). These data may suggest the expression of glutamine synthetase is more severely altered in cortex affected by severe neurodegeneration in Alpers’ syndrome.

Pooled SUDEP patient astrocytes harboured significantly decreased levels of glutamine synthetase in the occipital cortex relative to controls (*P* < 0.01). However, the SUDEP patient group also significantly differed to four of six patients with Alpers’ syndrome (*P* < 0.05). This may suggest altered glutamine synthetase protein expression is common in epilepsy, but appears to be more severe, albeit variable, in Alpers’ syndrome, which may be associated with more severe neurodegeneration.

### Increased phenotypic changes to occipital astrocytes

As a comparison, we also phenotypically characterised GFAP + astrocytes in frontal cortical tissues where neurodegeneration and gliosis are typically less severe in Alpers’ syndrome. However, patient occcipital and frontal cortical astrocytes were only directly compared when tissues were available for both cortical regions.

The frontal cortex from multiple patients with Alpers’ syndrome demonstrated increased GFAP + labelling relative to the control group (Supplementary Fig. 4a however, this was frequently less severe compared to the occipital cortex (Supplementary Fig. 4b), and frontal astrocytes were frequently significantly smaller than occipital astrocytes (Supplementary Fig. 5). Interestingly, group level analyses revealed no difference in the levels of frontal NDUFB8 and COXI protein deficiencies in the Alpers’ syndrome patient group compared to the control group (*P* > 0.05), and comparisons between individual patients with Alpers’ syndrome frequently revealed significantly decreased abundance of NDUFB8 (*P* < 0.01), and to a lesser extent COXI, proteins in occipital versus frontal cortical astrocytes (Supplementary Fig. 6). Furthermore, quantitative analysis of Kir4.1 and AQP4 in frontal cortical astrocytes demonstrated a significantly increased expression (*P* < 0.05) of these proteins in half of the Alpers’ syndrome patient cohort (Supplementary Fig. 7). However, comparison of Kir4.1 and AQP4 protein expression between occipital and frontal cortical patient astrocytes frequently revealed a significantly higher expression in occipital astrocytes (Supplementary Fig. 7). These data suggest reactive astrogliosis shows a predilection for the occipital cortex in Alpers’ syndrome, and phenotypic changes to key astrocytic and mitochondrial proteins are more severely altered in occipital versus frontal cortical astrocytes in Alpers’ syndrome.

Similar to the occipital cortex, the expression of glutamine synthetase was variable in frontal cortical astrocytes in the Alpers’ syndrome patient group involving a decreased intensity in four of seven patients with Alpers’ syndrome (*P* < 0.01) and increased intensity in one patient relative to control group (*P* < 0.0001) (Supplementary Fig. 7). It is possible altered (increased or decreased) levels of glutamine synthetase protein expression may reflect aberrant glutamate metabolism in Alpers’ syndrome.

## Discussion

The pathological mechanisms underpinning epilepsy in POLG-related disease remain obscure. However, the dysfunction of astrocytes is hypothesised to exacerbate the neuronal hyperexcitability resulting from the degeneration of inhibitory interneurons in POLG disease, thus contributing to the generation of seizure-associated activity [19, 22]. We therefore sought to phenotypically characterise changes to reactive astrocytes in post-mortem brain tissues from thirteen patients with Alpers’ syndrome. We have demonstrated changes to crucial astrocytic proteins involved in recycling neurotransmitters and buffering ions, supporting the contention that dysfunctional astrocytes have a role in the pathophysiology of Alpers’ syndrome.

Mitochondrial dysfunction due to a disproportionate loss of mitochondrial complex I within astrocytes has previously been reported in adult patients with mitochondrial epilepsy harbouring pathogenic variants in mtDNA and *POLG* [22]. Corroborating this finding, we have demonstrated a variably severe loss of mitochondrial complex I subunits, and a more subtle loss of mitochondrial complex IV subunits within reactive astrocytes from patients with Alpers’ syndrome. Notably, our analyses revealed OXPHOS protein deficiencies were more severe in astrocytes of the occipital cortex versus the frontal cortex in Alpers’ syndrome patient tissues, reflecting the severity of pathology observed in these cortices. Although astrocytes are reported to be predominantly glycolytic-dependent cells [48], knockdown of the mtDNA helicase Twinkle (*TWNK*) specifically within astrocytes of mice causes a spongiotic encephalopathy characterised by early onset neurodegeneration, suggesting a link between astrocytic mitochondrial function and neuronal protection [23]. Furthermore, the localisation of mitochondria to astrocytic processes, where metabolic demands are high, supports a critical role of astrocytic mitochondria for regulating synaptic activity through the uptake of neurotransmitters and buffering of ions [49–51]. These synaptic events are ATP-dependent, thus, the OXPHOS protein deficiencies observed in Alpers’ syndrome astrocytes may have a direct consequence on seizure activity.

Astrocytes from patients with Alpers’ syndrome frequently showed increased expression of Kir4.1, particularly within occipital cortex tissues. Since inwardly rectifying Kir channels composed of Kir4.1 subunits remove K^+^ ions from the synapse to reduce neuronal depolarisations [52], it might be speculated that increased Kir4.1 expression could represent a compensatory mechanism to enhance dissipation of excess K^+^ ions which accumulate during mitochondrial-associated seizures, and to facilitate the uptake of glutamate into astrocytes [53–55]. This appears to be a distinct phenotypic change to Alpers’ syndrome astrocytes, which may be a secondary consequence to impaired astrocytic mitochondrial function, and differs from SUDEP patient group astrocytes, which frequently demonstrated a loss of Kir4.1 protein expression. Decreased expression and function of Kir4.1 channels is a common pathological finding in TLE, particularly within sites of epileptogenesis, suggesting impaired astrocytic clearance of K^+^ promotes neuronal hyperexcitability [28, 29, 56, 57]. Since the expression of Kir4.1 is reported to be tightly coupled to AQP4 [31], it is perhaps unsurprising AQP4 protein levels were also frequently increased in astrocytes from patients with Alpers’ syndrome, particularly within the occipital cortex and regions of severe neurodegeneration. Increased AQP4 expression has also been observed in sclerotic hippocampi from patients with TLE, the neuropil of patients with focal cortical dysplasia, and some SUDEP patient cortical astrocytes of the current study, albeit to a lesser extent compared to Alpers’ syndrome patient astrocytes [28, 30, 58]. This suggests changes to AQP4 is a common phenotypic change in epilepsy and is likely to be closely associated with altered ionic homeostasis, increasing the propensity for seizures [32, 59].

Decreased expression and enzymatic activity of glutamine synthetase is a recognised pathological feature of TLE and is associated with increased neurodegeneration at epileptic foci [25–27]. Similar to adult patients with mitochondrial epilepsy [22], we have reported decreased glutamine synthetase protein expression in multiple patients with Alpers’ syndrome. There was also a trend towards a more severe decrease in glutamine synthetase protein expression in focal lesioned cortical tissues suggesting glutamate metabolism is more severely impaired in epileptic foci, similar to the observation in TLE. Since glutamine synthetase activity is critical for the deamination of glutamate to glutamine, decreased abundance of glutamine synthetase may be associated with impaired recycling of neurotransmitters leading to an excitotoxic accumulation of glutamate and decreased synthesis of GABA, lowering the seizure threshold [60–62]. Astrocytes from the SUDEP patient group also frequently demonstrated decreased levels of glutamine synthetase, supporting the pathological involvement of altered glutamine synthetase expression in epilepsy [63].

Interestingly, two patients with Alpers’ syndrome demonstrated increased levels of glutamine synthetase protein expression, including a patient who died in status epilepticus. This may reflect a subacute response to increased seizure activity that is subsequently downregulated with chronic epilepsy. The expression of glutamine synthetase has been reported to be transiently upregulated during early stages of epileptogenesis in in vivo rodent models of epilepsy [64, 65]. Increased glutamine synthetase expression may represent a compensatory response of reactive astrocytes to seizure-associated activity to metabolise excess glutamate that accumulates during seizures. However, consequently this may lead to increased synthesis of glutamate, thereby promoting epileptogenesis. Therefore, it is possible perturbed glutamate metabolism, through an abnormal increase or decrease in glutamine synthetase protein expression, may be pathological and contribute to disturbed neuronal network activity in Alpers’ syndrome.

Taken together, our neuropathological findings of severe reactive astrogliosis and altered expression of key astrocytic proteins corroborate a vulnerability of the primary visual cortex in Alpers’ syndrome. The abundance of hypertrophic reactive astrocytes displaying signs of mitochondrial dysfunction and altered levels of Kir4.1, AQP4 and glutamine synthetase proteins were more numerous in occipital versus frontal cortical tissues from patients with Alpers’ syndrome. Phenotypic changes to astrocytes were also frequently more severe in lesioned versus non-lesioned tissues. It is unknown whether the phenotypic changes to reactive astrocytes observed in occipital cortex tissues from patients with Alpers’ syndrome are secondary changes in response to seizure-associated activity, neural dysfunction and/or neurodegeneration, to ameliorate neuronal hyperexcitability as frontal cortical tissues which were affected by severe neuronal degeneration also demonstrated astrocytic abnormalities. It is also likely there is a pre-existing impaired function of astrocytes in Alpers’ syndrome due to primary mitochondrial dysfunction, perturbing the neuroprotective functions of astrocytes and reducing metabolic support for neurons, thereby promoting seizure activity and associated neurodegeneration. Insights from induced pluripotent stem cell (iPSC)-derived astrocytes carrying pathogenic variants in *POLG* certainly suggest primary impairment to astrocytic mitochondrial function is detrimental for neurons and causes astrocytes to adopt a pro-inflammatory reactive phenotype [66, 67].

### Study limitations

Although we have provided crucial evidence of astrocytic abnormalities in Alpers’ syndrome, these changes likely reflect end-stage pathology secondary to severe neurodegeneration. Thus, we were unable to make inferences about astrocytic changes which potentially drive early stages of disease pathogenesis. Furthermore, due to the rarity of Alpers’ syndrome, access to post-mortem brain tissues from patients with confirmed bi-allelic pathogenic variants in *POLG* were limited. Therefore, this post-mortem study included a small clinically- and neuropathologically-defined Alpers’ syndrome cohort. The lack of short-fixed brain tissues obtained from both the occipital and frontal cortices, per case, further limited the cohort for our immunofluorescence investigations. Finally, it is recognised that anti-GFAP antibodies only label a subset of astrocytes [68], however, the limited tissue availability prevented the inclusion of additional markers to identify individual astrocytes, albeit the majority of Alpers’ syndrome patient astrocytes were GFAP+. This also precluded investigations to explore changes to AQP4 in relation to the cerebral vasculature.

### Future directions

Since astrocytes appear to exhibit a pathological phenotype in post-mortem brain tissues from patients with Alpers’ syndrome, it is critical changes to astrocytic functions are interrogated in model systems of POLG-related pathology. While cultures of astrocytes harbouring bi-allelic pathogenic variants in *POLG* are useful for providing a mechanistic insight to primary impairment of astrocytic functions in response to mitochondrial dysfunction [66, 67], it is crucial *POLG*-astrocytes are co-cultured with relevant populations of neurons to better understand the relationship between astrocytic and neuronal dysfunction in Alpers’ syndrome, and to fully recapitulate the pathophysiology of POLG-related epilepsy. Elucidating the cross-talk between astrocytes and microglia in Alpers’ syndrome is also critical, since it is becoming increasingly recognised that microglia adopt a pro-inflammatory role in epilepsy and may promote the activation of dysfunctional astrocytes and seizure-associated activity [69–71]. Microgliosis is a common pathological feature accompanying reactive astrogliosis in models of mitochondrial disease [23, 72], and is prominent in post-mortem brain tissues from patients with POLG-related disease [6].

## Conclusions

We have demonstrated severe astrocytic abnormalities in post-mortem brain tissues from patients with Alpers’ syndrome, with a marked involvement of the occipital cortex. We hypothesise altered expression of key astrocytic and mitochondrial proteins may reflect altered functions of astrocytes, exacerbating neuronal hyperexcitability in Alpers’ syndrome. However, delineating the exact pathophysiological role of astrocytes in Alpers’ syndrome is critical to better understand the specific mechanisms underpinning POLG-related epilepsy. Bolstering the neuroprotective functions of glial cells may offer an opportunity to ameliorate neuronal hyperexcitability and increase metabolic support for neurons [73, 74], thereby preventing or delaying seizure-associated activity and neurodegeneration in Alpers’ syndrome.

## Electronic supplementary material

Below is the link to the electronic supplementary material.

Supplementary Material 1 - 7: Supplementary Figures 1 - 7. Supplementary Figure 1 - Quantification of GFAP+ labelling. Supplementary Figure 2 Altered mitochondrial mass in GFAP+ astrocytes in Alpers’ syndrome. Supplementary Figure 3 - Changes to astrocytic proteins in focal lesioned versus non- lesioned occipital cortex. Supplementary Figure 4 - Reactive astrogliosis in the frontal cortex in Alpers’ syndrome. Supplementary Figure 5 - Area of frontal cortical astrocytes. Supplementary Figure 6 - Mitochondrial oxidative phosphorylation protein deficiencies in frontal cortical astrocytes in Alpers’ syndrome. Supplementary Figure 7 - Altered expression of Kir4.1, AQP4 and glutamine synthetase in frontal cortical astrocytes in Alpers’ syndrome.

Supplementary Material 8: Supplementary Tables 1 - 4

## Data Availability

The datasets generated and/or analysed during the current study are not publicly available but are available from the corresponding author on reasonable request.

## Abbreviations

AQP4: Aquaporin-4
COXI: Cytochrome *c* oxidase I
GFAP: Glial fibrillary acidic protein
mtDNA: Mitochondrial DNA
NDUFB8: NADH:ubiquinone oxidoreductase subunit B8
POLG: DNA polymerase gamma (Pol γ)
OXPHOS: Oxidative phosphorylation
SUDEP: Sudden unexpected death in epilepsy
TLE: Temporal lobe epilepsy
