# Supplementary material for: Astrocytic pathology in Alpers’ syndrome
Source: Acta Neuropathol Commun. 2023 May 31;11:86. doi: 10.1186/s40478-023-01579-w (PMC10230702; doi:10.1186/s40478-023-01579-w)
Supplement: Supplementary file 1 — Supplementary Material 1 - 7: Supplementary Figures 1 - 7. Supplementary Figure 1 - Quantification of GFAP+ labelling. Supplementary Figure 2 Altered mitochondrial mass in GFAP+ astrocytes in Alpers’ syndrome. Supplementary Figure 3 - Changes to astrocytic proteins in focal lesioned versus non- lesioned occipital cortex. Supplementary Figure 4 - Reactive astrogliosis in the frontal cortex in Alpers’ syndrome. Supplementary Figure 5 - Area of frontal cortical astrocytes. Supplementary Figure 6 - Mitochondrial oxidative phosphorylation protein deficiencies in frontal cortical astrocytes in Alpers’ syndrome. Supplementary Figure 7 - Altered expression of Kir4.1, AQP4 and glutamine synthetase in frontal cortical astrocytes in Alpers’ syndrome. [file 40478_2023_1579_MOESM1_ESM.pdf]

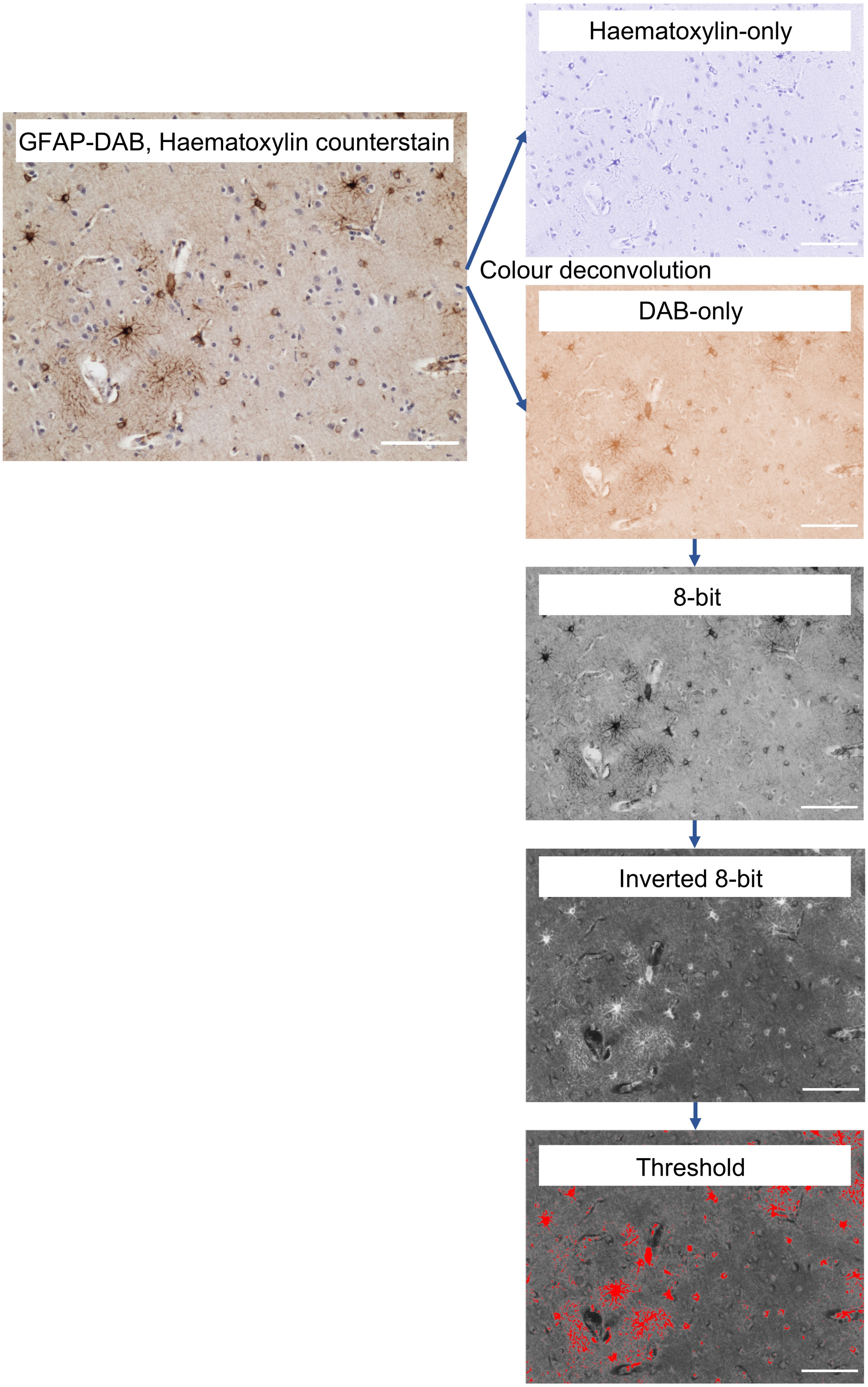

SUPPLEMENTARY FIGURE 1 Quantification of GFAP+ labelling. DAB-positive signals were differentiated from the Haematoxylin counterstain using a colour deconvolution plugin installed in FIJI (40). Images were then converted to 8-bit and were inverted (white = DAB+ stain). An intensity threshold was set to automatically detect DAB-positive signals (red = DAB) which was used to calculate the percentage area of tissue stained with GFAP (GFAP+ labelling).

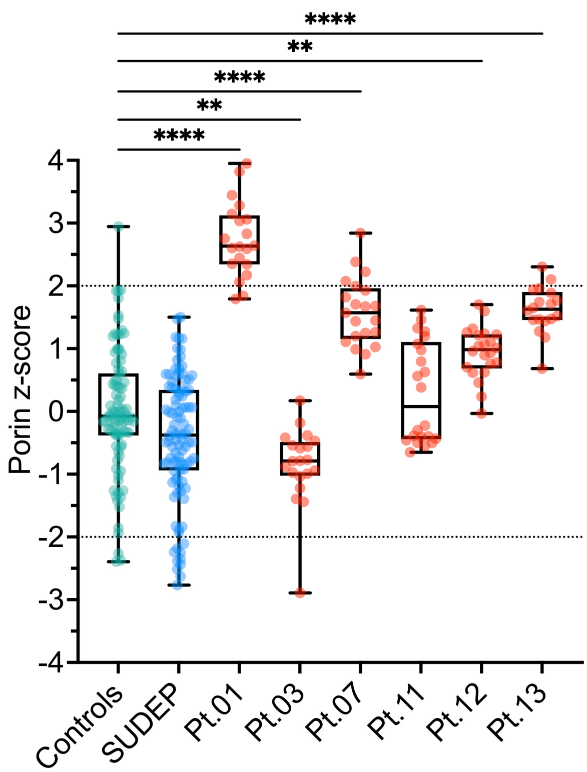

SUPPLEMENTARY FIGURE 2 Altered mitochondrial mass in GFAP+ astrocytes in Alpers' syndrome. The mean optical intensity of porin (mitochondrial marker) was measured within individual GFAP+ astrocytes of occipital cortex tissues. Z-scores were calculated to make inferences to mitochondrial mass in Alpers' syndrome: z-score > 2 = increased mitochondrial mass. Multiple comparison analyses relative to control data: * $P < 0.05$, ** $P < 0.01$, *** $P < 0.001$, **** $P < 0.0001$.

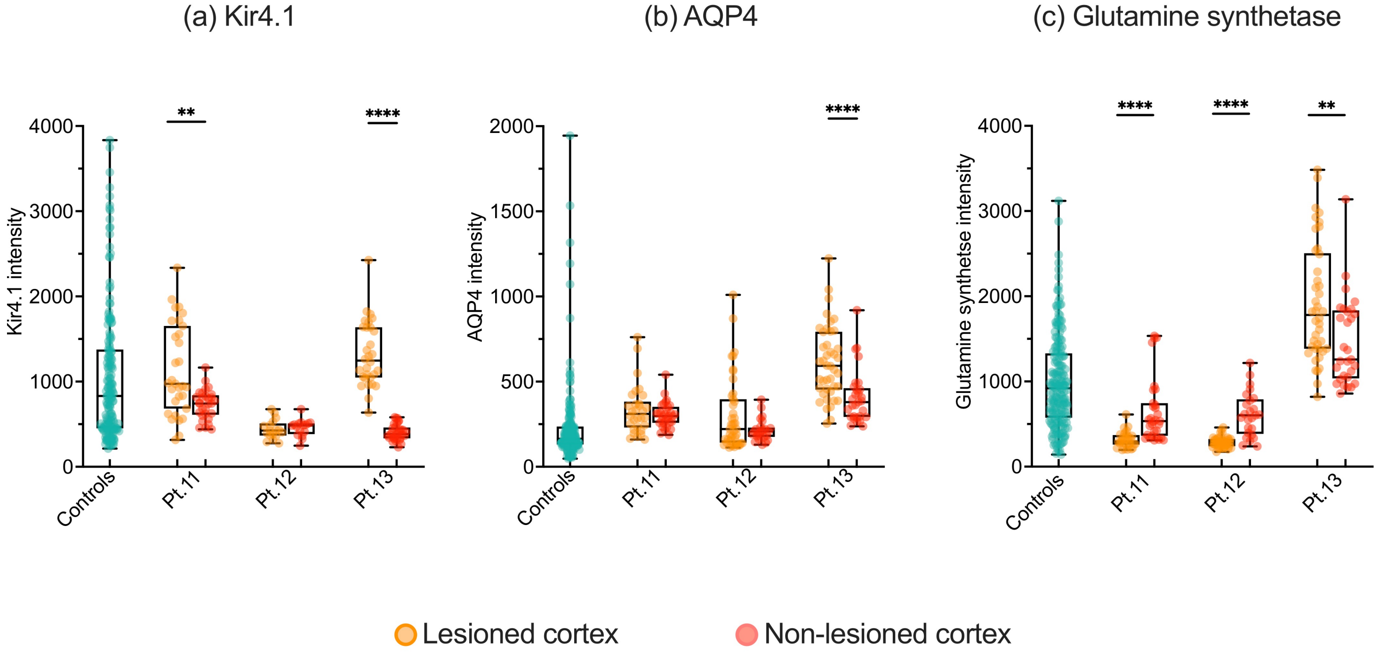

SUPPLEMENTARY FIGURE 3 Changes to astrocytic proteins in focal lesioned versus non-lesioned occipital cortex. The intensity of **(a)** Kir4.1, **(b)** AQP4 and **(c)** glutamine synthetase were quantified within GFAP+ astrocytes in demarcated focal lesioned occipital cortex (orange) and adjacent non-lesioned occipital cortex (red) of Pt.11, Pt.12 and Pt.13. Focal lesioned cortex was defined as a demarcated region of cortical tissue affected by almost total neuronal loss and severe gliosis. Patient data analysed using Mann-Whitney test: ** $P < 0.01$, **** $P < 0.0001$.

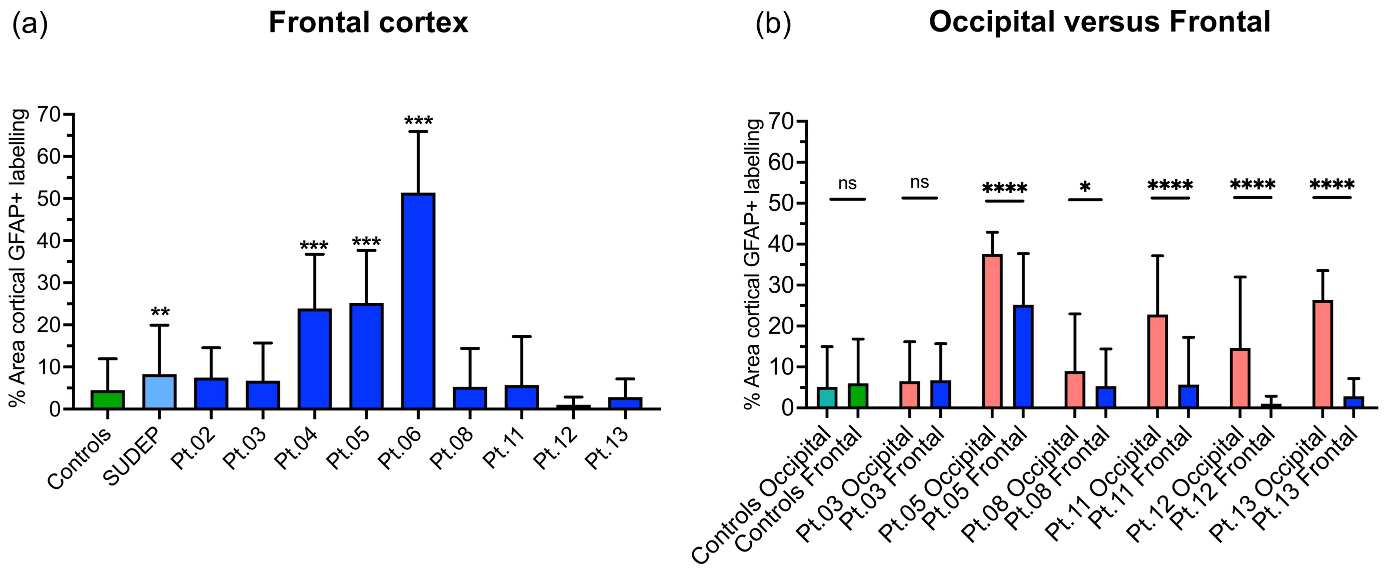

SUPPLEMENTARY FIGURE 4 Reactive astrogliosis in the frontal cortex in Alpers' syndrome. **(a)** Quantitative analysis of the percentage area of cortical GFAP+ staining revealed significantly increased GFAP+ labelling in multiple Alpers' syndrome patient tissues compared to controls in the frontal cortex (N=7) (40). Data presented as mean +SD. Multiple comparison analyses relative to control data: *** $P < 0.001$. **(b)** Data analysed using Mann-Whitney *U* test to compare the percentage area of GFAP+ labelling between the occipital and frontal cortex for the control group and patients with Alpers' syndrome for which tissues from both cortical regions were available: * $P < 0.05$, **** $P < 0.0001$, *ns* $P > 0.05$.

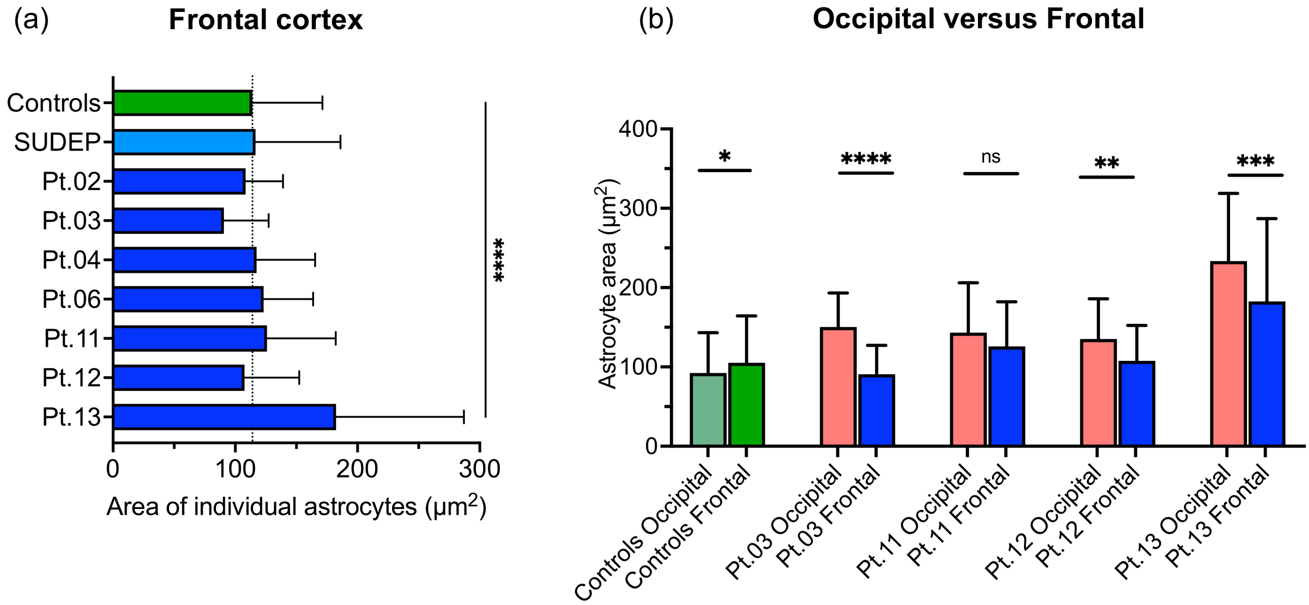

SUPPLEMENTARY FIGURE 5 Area of frontal cortical astrocytes. (a) The mean (+ SD) area (μm^2) of individual two-dimensional (2-D) GFAP+ astrocytes in frontal cortical tissues. Dotted line indicates the mean area of control astrocytes. Multiple comparison analyses relative to control data: **** $P < 0.0001$. **(b)** Data analysed using Mann-Whitney *U* test to compare the area (μm^2) of occipital versus frontal cortical GFAP+ astrocytes for the control group and patients with Alpers' syndrome for which tissues from both cortical regions were available. Analyses revealed occipital cortical astrocytes are frequently larger than frontal cortical astrocytes in Alpers' syndrome. * $P < 0.05$, ** $P < 0.01$, *** $P < 0.001$, **** $P < 0.0001$, *ns* $P > 0.05$.

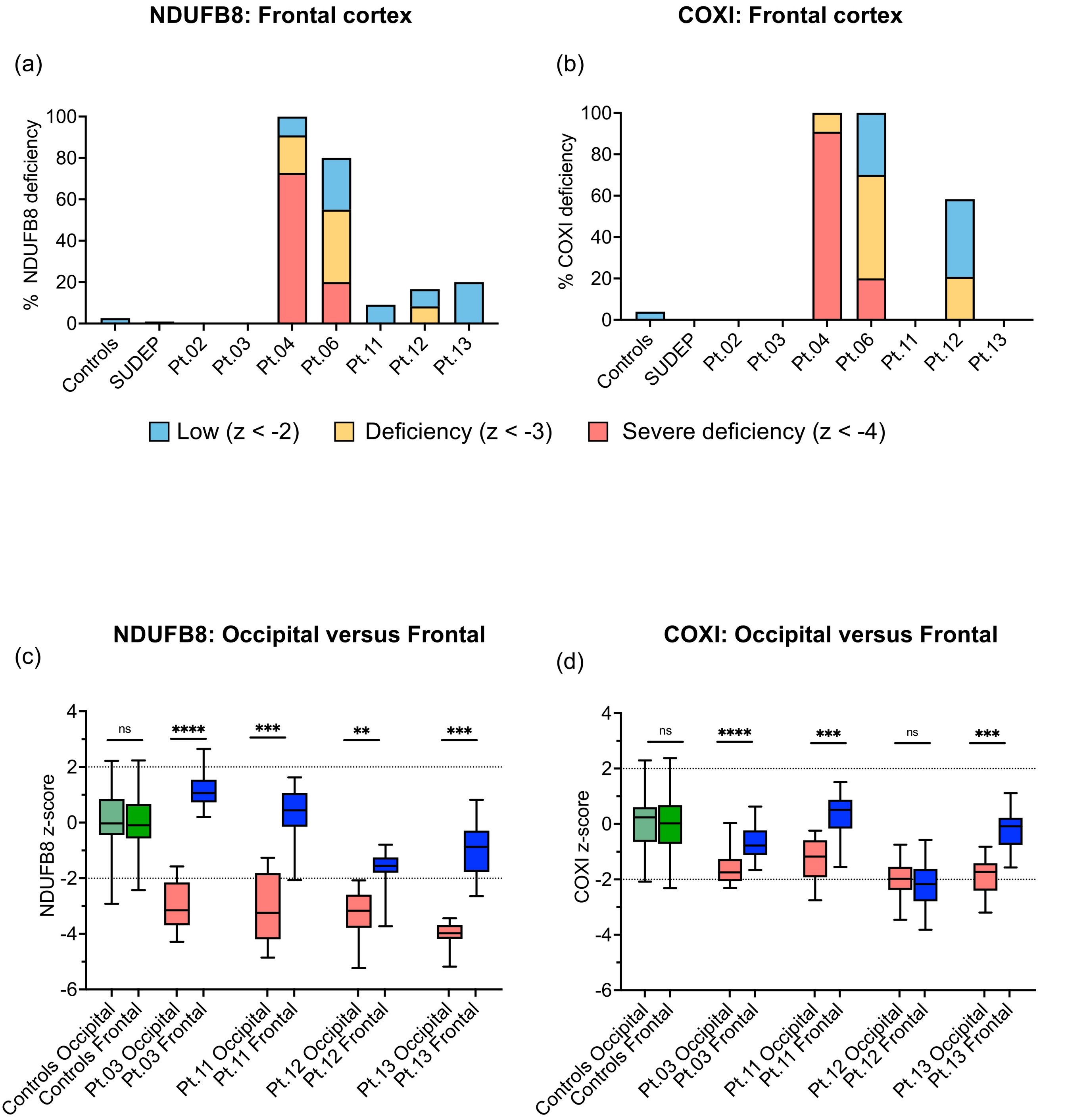

SUPPLEMENTARY FIGURE 6 Mitochondrial oxidative phosphorylation protein deficiencies in frontal cortical astrocytes in Alpers' syndrome. (a – b) The percentage of GFAP+ astrocytes with decreased mean optical intensity of NDUFB8 **(a)** and COXI **(b)** normalised to porin, relative to control astrocytes. Levels of deficiencies are based on standard deviation limits of control data: z-score < -2 = low expression; z-score < -3 = deficient; z-score < -4 = severely deficient (45). N=4 Controls, N=5 SUDEP cases. **(c – d)** Comparison of the levels of NDUFB8 **(c)** and COXI **(d)** protein deficiencies in occipital versus frontal cortical astrocytes for the control group and patients with Alpers' syndrome for which tissues were available for both cortical regions. Mann-Whitney *U* test revealed NDUFB8 and COXI protein deficiencies were frequently more severe in occipital versus frontal cortical astrocytes in Alpers' syndrome: ** $P < 0.01$, *** $P < 0.001$, **** $P < 0.0001$, *ns* $P > 0.05$.

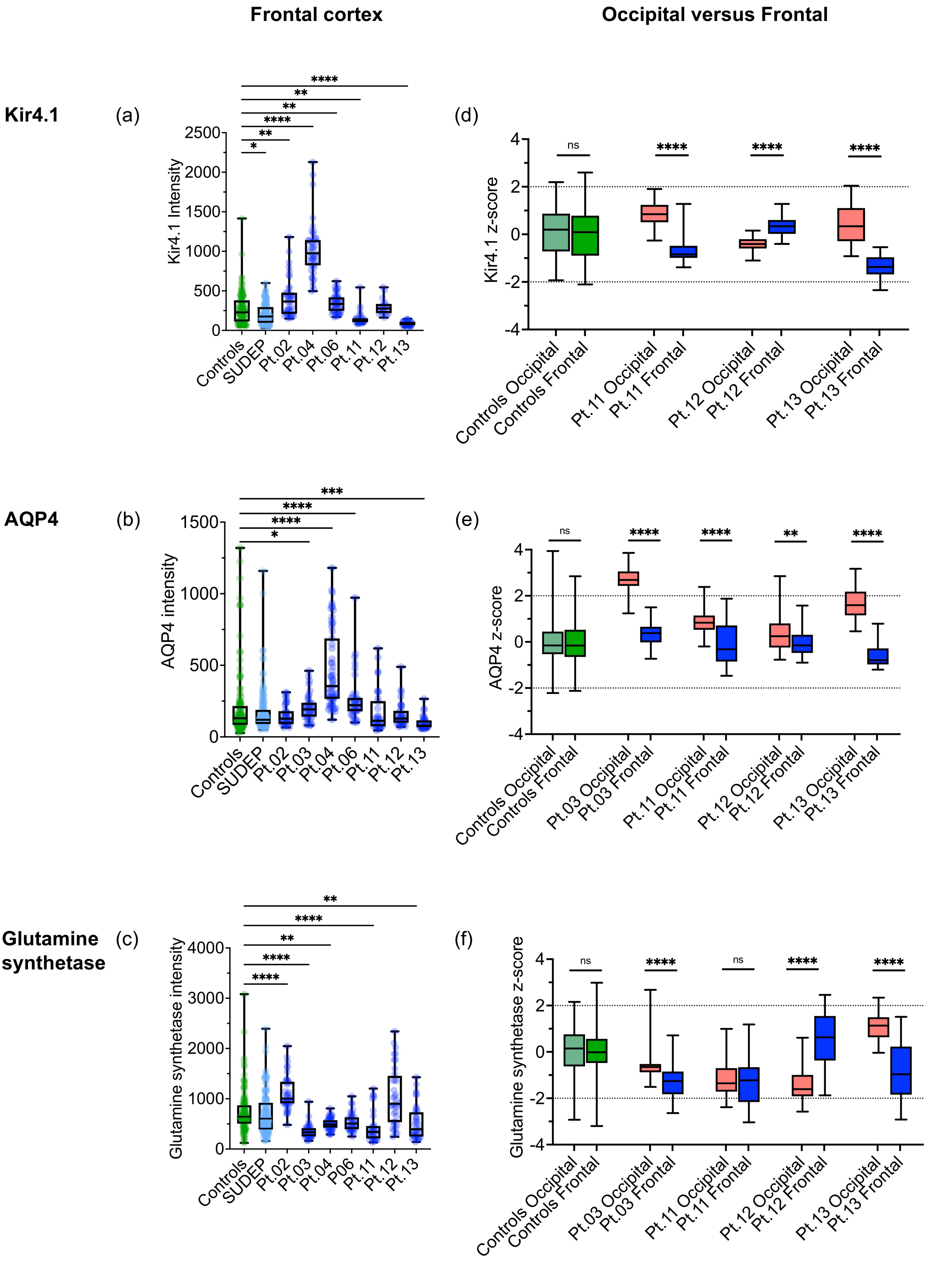

SUPPLEMENTARY FIGURE 7 Altered expression of Kir4.1, AQP4 and glutamine synthetase in frontal cortical astrocytes in Alpers' syndrome. Multiple comparison analyses demonstrate the intensity of Kir4.1 **(a)**, AQP4 **(b)** and glutamine synthetase **(c)** in Alpers' syndrome patient astrocytes compared to control astrocytes: **P* < 0.05, ** *P* < 0.01, *** *P* < 0.001, **** *P* < 0.0001. **(d – f)** Occipital astrocytes were compared to frontal astrocytes using the Mann-Whitney *U* Test when patient tissues were available for both cortical regions: ** *P* < 0.01, **** *P* < 0.0001, *ns P* > 0.05.
