# Supplementary material for: Astrocytic pathology in Alpers’ syndrome
Source: Acta Neuropathol Commun. 2023 May 31;11:86. doi: 10.1186/s40478-023-01579-w (PMC10230702; doi:10.1186/s40478-023-01579-w)
Supplement: Supplementary file 2 — Supplementary Material 8: Supplementary Tables 1 - 4 [file 40478_2023_1579_MOESM2_ESM.docx]

**Supplementary Table 1**: Demographic details for the patient and control cohort

| ***Patient:*** | ***Sex:*** | ***Age at death:*** | ***Brain bank:*** | ***Post-mortem  interval: (hours)*** | ***Formalin fixation duration:*** | ***Cause of death:*** | ***Occipital tissue:*** | ***Frontal tissue:*** |
| --- | --- | --- | --- | --- | --- | --- | --- | --- |
| Pt.01 | M | 5.5 m | Vienna | Unknown | Unknown | Cardiac and respiratory failure | Yes | No |
| Pt.02 | M | 13 m | Vienna | Unknown | Unknown | Pneumonia | No | Yes |
| Pt.03 | F | 14 m | Newcastle Brain Tissue Resource | 12 | 1 m | Hepatic failure | Yes | Yes |
| Pt.04 | M | 17 m | Southampton* | Unknown | 6 m | Unknown | No | Yes |
| Pt.05 | F | 18 m | NeuroBioBank | 17 | 18.8y | Respiratory failure | Yes | Yes |
| Pt.06 | M | 2.8 y | Oxford* | Unknown | 4 m | Respiratory failure | No | Yes |
| Pt.07 | F | 7 y | Vienna | Unknown | Unknown | Pneumonia | Yes | No |
| Pt.08 | M | 11.9 y | NeuroBioBank | 21 | 9 y | Complication of disorder | Yes | Yes |
| Pt.09 | M | 12.5 y | Oxford* | Unknown | 32 y | Uncontrollable myoclonic epilepsy | Yes | No |
| Pt.10 | F | 14 y | Oxford* | 48 | 17 y | Unknown | Yes | No |
| Pt.11 | F | 23 y | Newcastle Brain Tissue Resource | 32 | 1 m | Status epilepticus | Yes | Yes |
| Pt.12 | F | 24 y | Newcastle Brain Tissue Resource | 83 | 4.5 m | Suppurative tracheobronchitis | Yes | Yes |
| Pt.13 | F | 28 y | Newcastle Brain Tissue Resource | 64 | 1 m | Status epilepticus | Yes | Yes |
| S.01 | F | 17 m | Oxford* | 120 | <1 y | Sudden unexpected death in epilepsy | Yes | Yes |
| S.02 | F | 4.5 y | Oxford* | 48 | <5 m | Sudden unexpected death in epilepsy | Yes | Yes |
| S.03 | M | 10 y | Oxford* | 144 | <9 m | Sudden unexpected death in epilepsy | Yes | Yes |
| S.04 | M | 19 y | Newcastle Brain Tissue Resource | Unknown | Unknown | Sudden unexpected death in epilepsy | Yes | Yes |
| S.05 | M | 27 y | Oxford* | 48 | <4 m | Sudden unexpected death in epilepsy | Yes | Yes |
| C.01 | F | 14 m | NeuroBioBank | 20 | 3.1 y | Coarctation of aorta | Yes | Yes |
| C.02 | M | 12.7 y | NeuroBioBank | 15 | 9.3 y | Hanging / suicide | Yes | Yes |
| C.03 | F | 14.5 y | NeuroBioBank | 8 | 9 y | Streptococcal toxic shock syndrome | Yes | Yes |
| Ct.04 | M | 16 y | Oxford* | 72 | 1 m | Unascertained sudden death | Yes | No |
| Ct.05 | M | 16 y | Edinburgh Brain and Tissue Bank | 47 | 4 d | Suspension by ligature | Yes | Yes |
| Ct.06 | F | 16 y | Edinburgh Brain and Tissue Bank | 49 | 6 d | Sudden cardiac death | Yes | Yes |
| Ct.07 | F | 18 y | Newcastle Brain Tissue Resource | 81 | 6 m | MDMA toxicity, cardiac arrest | Yes | Yes |
| Ct.08 | F | 24 y | Edinburgh Brain and Tissue Bank | 47 | 9 d | Suspension by ligature | Yes | Yes |

Alpers’ syndrome patients (Pt.01 – Pt.13); sudden unexpected death in epilepsy patients (S.01 – S.05); controls (C.01 – C.08). Tissues fixed for a known duration of more than 1 year (y) were not included for immunofluorescence experiments. * BRAIN UK Centres in Southampton and Oxford.

**Supplementary Table 2**: Clinical summaries

| ***Patient ID:*** | ***Age at death:*** | ***History of  refractory  epilepsy:*** | ***EEG findings:*** | ***Evidence of stroke-like episodes:*** | ***Patient ID in Smith et al., 2022.*** |
| --- | --- | --- | --- | --- | --- |
|  |  |  |  |  |  |
| Pt.01^†^ | 5.5 m | Yes | Slow base rhythm, frontal delta waves, generalised spikes |  | P01 |
| Pt.02^†^ | 13 m | Yes | Hypsarrhythmia |  | P02 |
| Pt.03 | 14 m | Yes | Widespread irregular alpha and theta, moderate delta activity |  | P03 |
| Pt.04^†^ | 17 m | Yes | Unknown |  | P04 |
| Pt.05^†^ | 18 m | Yes | Unknown |  | P05 |
| Pt.06^†^ | 2.8y | Yes | Unknown |  | P06 |
| Pt.07 | 7 y | Yes | Occipital slow spike wave variants; dominant delta activity in fronto-parietal regions; asymmetric dysrhythmia. |  | P08 |
| Pt.08 | 11.9 y | Yes | Posterior dominant rhythm of 5 Hz over both occipital lobes. Frequent posterior intermittent delta polymorphic activity of 2.5-3 Hz. |  | P09 |
| Pt.09^†^ | 12.5 y | Yes | Widespread irregular slow wave activity |  | P10 |
| Pt.10^†^ | 14 y | Yes | Abnormal isoelectric activity |  | P11 |
| Pt.11 | 23 y | Yes | Continuous right posterior spike-and-wave activity | Occipital | P12 |
| Pt.12 | 24 y | Yes | Encephalopathic, right posterior quadrant waves | Occipital, parietal, thalamic | P13 |
| Pt.13 | 28 y | Yes | Unknown | Occipital, frontal, parietal | P14 |

^†^ Denotes historical patients for which clinical details are limited. Stroke like episodes confirmed radiologically on magnetic resonance imaging (MRI). Electroencephalogram (EEG) and MRI findings for this patient cohort have previously been published in ([19](#_ENREF_19)).

**Supplementary Table 3**: Primary antibodies

| ***Antibody*** | ***Target*** | ***Host isotype*** | ***Antigen retrieval*** | ***Antibody dilution for immunofluorescence*** *(immunohistochemistry)* | ***Antibody supplier*** | ***Catalogue number*** |
| --- | --- | --- | --- | --- | --- | --- |
| **Glial fibrillary protein** | Reactive astrocytes | Mouse IgG1 | Citrate | 1:50 | DAKO | M0761 |
|  |  | Rabbit IgG | Citrate or EDTA | 1:2000  (1:15,000 immunohistochemistry) | DAKO | Z0334 |
| **NDUFB8** | Mitochondrial complex I subunit | Mouse IgG1 | EDTA | 1:100 | Abcam | Ab110242 |
| **COXI** | Mitochondrial complex IV subunit | Mouse IgG2a | EDTA | 1:200 | Abcam | Ab14705 |
| **Porin** | Mitochondrial membrane pore | Mouse IgG2b | EDTA | 1:200 | Abcam | Ab14734 |
| **Kir4.1** | Astrocytic potassium ion channel subunit | Rabbit IgG | Citrate | 1:100 | Alomone | APC-035 |
| **Aquaporin 4 (AQP4)** | Astrocytic water channel | Rabbit IgG | Citrate | 1:3000 | Merck | A5971 |
| **Glutamine synthetase** | Astrocytic glutamate metabolising enzyme | Mouse IgG2a | Citrate | 1:6000 | BD Transduction Laboratories | 610518 |

**Antigen retrieval methods***:* 1mM EDTA (pH 8.0) in a pressure cooker for 40 minutes, or 10mM Trisodium citrate (pH 6.0) microwaved for 10 minutes followed by 10 minutes cooling.

**Immunohistochemistry:** Menarini Diagnostics horseradish-peroxidase polymer kit was used for primary antibody amplification which was visualised using DAB (MenaPath X-Cell Plus HRP-polymer kit, A. Menarini diagnostics)

**Blocking**: 3% hydrogen peroxide (H_2_O_2_, diluted in H_2_O) block for 15 minutes at room temperature for immunohistochemistry experiments, or 10% normal goat serum (NGS, diluted in TBST) block for 1 hour at room temperature for immunofluorescence experiments. Sections stained with NDUFB8 were also blocked in avidin for 15 minutes, followed by blocking in biotin for 15 minutes at room temperature.

**Supplementary Table 4**: Secondary antibodies

| ***Secondary antibody*** | ***Target  (primary antibody)*** | ***Host*** | ***Dilution*** | ***Supplier*** | ***Catalogue number*** |
| --- | --- | --- | --- | --- | --- |
| Alexa Fluor 405 | Rabbit IgG  (GFAP) | Goat IgG | 1:100 (2 hours 4^o^C) | ThermoFisher Scientific | A31556 |
| Alexa Fluor 488 | Mouse IgG2a (Glutamine synthetase or COXI) |  |  |  | A21131 |
|  | Mouse IgG1  (GFAP) |  |  |  | A21121 |
| Alexa Fluor 546 | Mouse IgG1  (GFAP) |  |  |  | A21123 |
| Alexa Fluor 647 | Rabbit IgG  (AQP4, Kir4.1) |  |  |  | A21242 |
|  | Mouse IgG2b  (Porin) |  |  |  | A21244 |
| Alexa Fluor 546-streptavidin | Biotin |  |  |  | S11225 |
| Biotin | Mouse IgG1 (NDUFB8) |  | 1:200 (room temperature) |  | A10519 |

**Multiplex immunofluorescence experiments:**

**(1)** GFAP (405nm), COXI (488nm), NDUFB8 (546nm), Porin (647nm)

**(2)** Hoechst, Glutamine synthetase (488nm), GFAP, (546nm), AQP4 (647nm)

**(3)** Hoechst, GFAP (488nm), Kir4.1 (647nm)
